# Antigen-Specific Immunotherapy for Treatment of Autoimmune Liver Diseases

**DOI:** 10.3389/fimmu.2020.01586

**Published:** 2020-07-21

**Authors:** Naomi Richardson, Sky T. H. Ng, David C. Wraith

**Affiliations:** Institute of Immunology and Immunotherapy, College of Medical and Dental Sciences, University of Birmingham, Birmingham, United Kingdom

**Keywords:** immunoregulation, liver, autoimmune disease, immunotherapy, T-cell

## Abstract

The liver is a critical organ in controlling immune tolerance. In particular, it is now clear that targeting antigens for presentation by antigen presenting cells in the liver can induce immune tolerance to either autoantigens from the liver itself or tissues outside of the liver. Here we review immune mechanisms active within the liver that contribute both to the control of infectious diseases and tolerance to self-antigens. Despite its extraordinary capacity for tolerance induction, the liver remains a target organ for autoimmune diseases. In this review, we compare and contrast known autoimmune diseases of the liver. Currently patients tend to receive strong immunosuppressive treatments and, in many cases, these treatments are associated with deleterious side effects, including a significantly higher risk of infection and associated health complications. We propose that, in future, antigen-specific immunotherapies are adopted for treatment of liver autoimmune diseases in order to avoid such adverse effects. We describe various therapeutic approaches that either are in or close to the clinic, highlight their mechanism of action and assess their suitability for treatment of autoimmune liver diseases.

## Immunology of the Liver, an Overview

The liver is a complex immune-rich organ with a propensity toward tolerance, central to its role in blood filtration and toxin removal. This characteristic is most striking in cases of successful liver transplantation in which patients can be safely weaned off immunosuppression and in multi-organ transplants where transplanting liver alongside other organs including lung and heart prevents multi-organ rejection (1–4).

**Figure 1:**
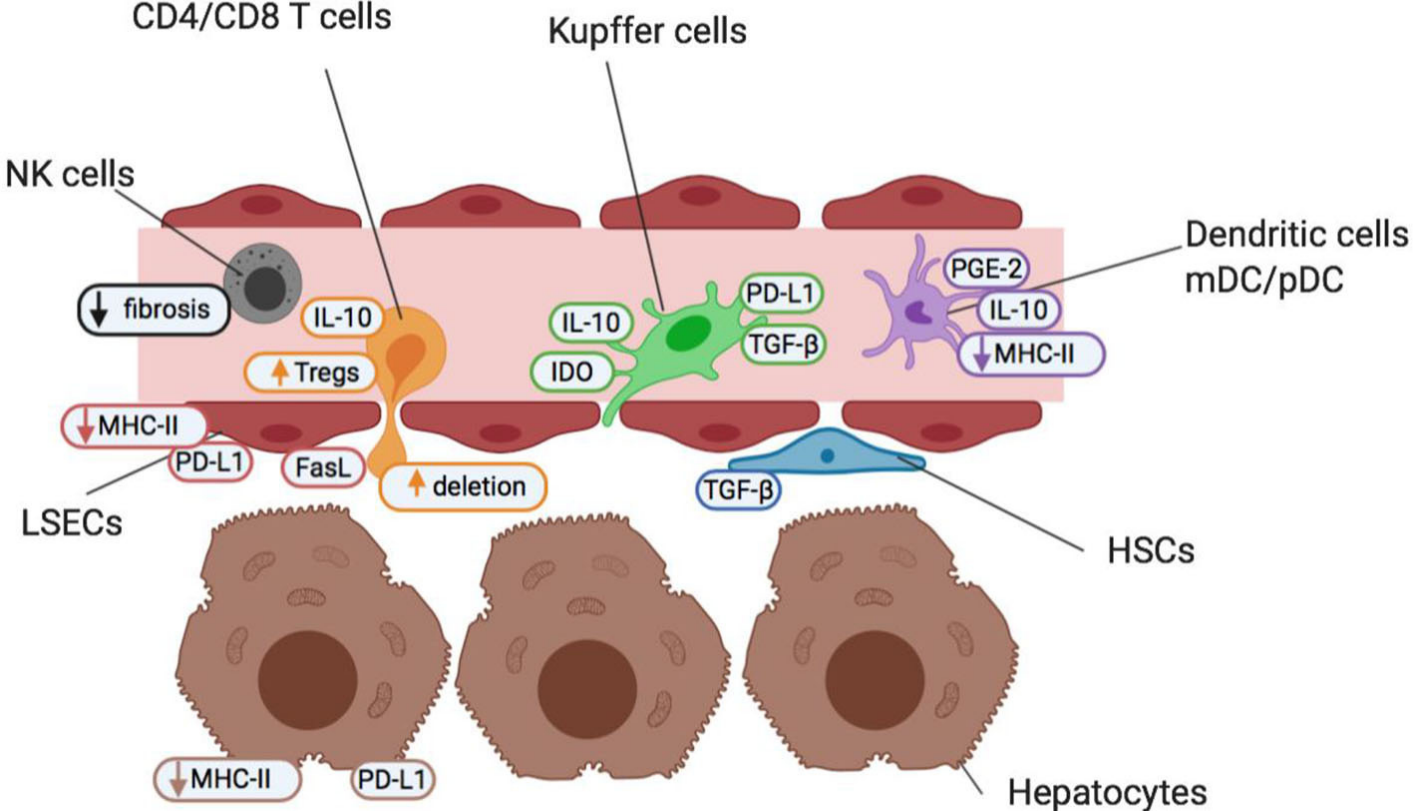
Cells of the liver sinusoid environment and their functions help maintain a state of homeostatic tolerance in the liver. Non-parenchymal resident liver cells including Kupffer cells (green), hepatic stellate cells (HSCs; blue), liver sinusoidal endothelial cells (LSECs; red) and dendritic cells (myeloid mDC and plasmacytoid pDC; purple) are situated within, or in close proximity to, liver sinusoids forming an early detection system to identify pathogens and maintain barrier function. They contribute to the maintenance of a high anti-inflammatory TGF-β and IL-10 cytokine milieu under steady-state conditions and in the face of common bacterial and food antigens to which the liver is continuously exposed. The liver also contains high numbers of innate-like immune cells such as NK cells (gray), and δT cells (not shown). NK cells act as pro-inflammatory agents, and promote the recruitment of effector immune cells, but are also key regulators of fibrosis. Both non-parenchymal antigen-presenting cells and hepatocytes (brown) offer a reduced antigen-presentation capacity and lower levels of costimulation than other antigen-presenting cells elsewhere in the body. This helps promote an environment of low T cell (orange) activation under normal conditions and maintain a state of “active” tolerance, whereby if required, inflammation and T cell activation is readily engaged.

As the liver receives both arterial blood and blood from the gut via the portal vein, it is regularly exposed to both dietary and microbial antigens, which could establish excessive and prolonged inflammation, tissue damage and fibrosis if unregulated. Therefore, diverse populations of immune cells, stromal cells and hepatocytes work in synergy to resolve localized inflammation and avoid unnecessary immune responses to innocuous stimuli (5, 6). The liver microenvironment is well-adapted to maintain homeostasis due to its unique populations of antigen-presenting cells (APC) with tolerogenic characteristics, feedback mechanisms to control inflammation, high density of innate immune cells and richness of suppressive soluble mediators (summarized in Figure 1 and Table 1).

**Table 1:** Summary of tolerogenic functions exerted by non-parenchymal liver cells and hepatocytes and their physiological effects.

**Cell type**	**Mechanisms**	**Effects**	**References**
NK	Become cytotoxic in response to IL-18 and TRAIL receptor ligation	Direct and indirect killing of activated HSCs	(7, 8)
DCs (myeloid and plasmacytoid)	Expression of low MHC-II and costimulatory molecules CD80/CD86 and CD40, low secretion of IL-12	Poor T cell priming - induction of anergy or deletion of antigen-specific T cells. Poor differentiation of naïve CD4+ T cells to Th1 effector cells	(9, 10)
	Secretion of IL-10	Bias toward generation of CD25+FoxP3+ Tregs and Th2 cells Reduced production of pro-inflammatory cytokines TNF-α, IL-6 and ROS by monocytes	(9)
	Production of PGE-2	Inhibition of T cell proliferation and induces apoptosis, induction of regulatory dendritic cells	(11)
	Expression of PD-L1	Inhibition of T cell activation and induction of apoptosis of activated T cells	(12)
Tregs	Production of IL-10	Downregulation of CCR7 on liver DCs preventing their recirculation to secondary lymphoid tissues	(13)
LSECs	Production of PGE2 and IL-10	Inhibition of T cell proliferation, decreased pro-inflammatory cytokine production, increased Treg generation	(14, 15)
	Cross-presentation of antigen to CD8+ T cells	CD8+ T cells are rendered unresponsive, preferential deletion when PD-1/PD-L1 engaged	(16, 17)
	Expression of PD-L1	Inhibition of T cell activation and induction of apoptosis of activated T cells	(18)
	Expression of FasL	Allospecific T cells crossing LSEC barrier undergo apoptosis	(19)
	Expression of low MHC-II and costimulatory molecules CD80/CD86 and CD40	Poor T cell priming - naïve CD4 do not effectively differentiate to Th1 effector cells. Th1 and Th17 cells lose effector potency in contact with LSECs	(18)
Kupffer cells	Production of IDO, PGE2, TGF-β and IL-10	Reduced production of pro-inflammatory cytokines TNF-α, IL-6, increased Treg generation	(20)
	Low expression of MHC-II, CD80, CD86, and CD40	Poor direct T cell priming - naïve CD4 do not effectively differentiate to effector cells	(21)
	Production of prostaglandins	Inhibit dendritic cells priming of T cells, reduced Th1 and Th17 output	(21, 22)
	Scavengers of antigen at steady-state	Induce/maintain T cell tolerance to antigen by expansion of IL-10 producing Tregs and arrest of CD4+ Tconv	(22)
HSCs	Expression of PD-L1 and TRAIL when activated	Inhibition of T cell activation and induction of TRAIL-mediated apoptosis	(23, 24)
	Production of TGF-β and retinoic acid	Increased Treg differentiation	(25, 26)
Hepatocytes	MHC-II expression with very low expression of costimulatory molecules	Poor T cell priming - induction of anergy or deletion of antigen-specific T cells	(27, 28)
	Expression of PD-L1	Inhibition of T cell activation and induction of apoptosis of activated T cells	(29, 30)
	Activation of Notch signaling pathway on Th1	Diverts Th1 CD4+ T cells to synthesize IL-10	(31)

For example, cells of the hepatic sinusoids are continuously exposed to Gram-negative bacterial endotoxin e.g., lipopolysaccharide (LPS), which is detectable in portal vein blood but not systemic circulation (32). These cells when engaging with LPS via Toll-like receptor 4 (TLR4) are adapted to have an increased activating threshold to avoid hyper-active signaling and to better remove LPS from the blood stream (33).

### Innate Immune Cells in the Liver

The liver is enriched for innate immune cells which help trigger strong activating signals for inflammation in situations where tolerance is unsuitable, e.g., pathogen infection. Around 50% of liver resident lymphocytes are NK cells (Figure 1, gray), notably higher than in most tissues (34). Similarly, numbers of unconventional T cells, NK-T and γδT cells, are increased in the liver to recognize lipid antigens and bacterial pathogens, respectively (35, 36). Activated NK and NKT cells produce significant amounts of cytokines, including strongly inflammatory TNF-α and GM-CSF in response to viral and bacterial pathogens, to shift the balance from tolerance to inflammation. Activated liver NK cells produce IFN-γ and exert cytotoxicity due to TRAIL receptor binding and in response to IL-18 released by Kupffer cells (7, 8). Intriguingly, cytotoxic NKs also contribute to prevention of fibrosis by IFN-γ dependent arrest and apoptosis of hepatic stellate cells (HSCs) as well as directly killing activated HSC (37, 38). The role of γδT cells in the liver is currently less well-defined, but they are known to accumulate in both human fibrotic liver and experimental liver injury models and are producers of IL-17 (39, 40).

### Antigen-Presentation in the Liver

The liver is home to a wide range of APC with a tolerogenic bias, including liver sinusoidal endothelial cells (LSECs; Figure 1, red), resident myeloid and plasmacytoid dendritic cells (mDCs and pDCs; Figure 1, purple), Kupffer cells (KCs; Figure 1, green) and hepatic stellate cells (HSCs; Figure 1, blue). Antigen-presentation and costimulatory capacity of resting APC in the liver is generally low, contributing to the liver's state of active tolerance.

#### Dendritic Cells

Mouse and human liver resident DCs are tolerogenic under steady-state conditions, as they display a more immature phenotype with significantly lower expression of MHC Class II and CD80/CD86 than DCs found elsewhere (9). When activated by TLR4 ligands, liver DCs produce substantial amounts of anti-inflammatory prostaglandin E2 (PGE2) (11) and IL-10 whereas blood DCs produce almost exclusively inflammatory cytokines. Therefore, liver DCs are less capable to provide sufficiently strong signals required to activate T cells. Instead, DC-T cell interactions generate more CD25+FoxP3+ Tregs and IL-4 producing Th2 cells by an IL-10 dependent mechanism (9). IL-10 also downregulates the expression of CCR7 on circulating DCs preventing their re-circulation to secondary lymphoid tissue (13).

#### Liver Sinusoidal Endothelial Cells

LSECs express both MHC-I and MHC–II, and are as capable at antigen-uptake as DCs (41). They can, therefore, prime CD4+ T cells and cross-present antigen to CD8+ T cells, a function which is modulated by liver IL-10 (14). In both cases, the interaction between LSEC and T cell is biased toward tolerance. Naïve CD4 T cells primed by LSECs do not receive high costimulation, or an IL-12 stimulus from neighboring tolerogenic DCs and, therefore, do not effectively differentiate to Th1 effector cells (42–44). Th1 and Th17 cells when in contact with tolerogenic LSECs are unable to produce high levels of IFN-γ and IL-17, respectively (18). LSECs constitutive expression of PDL-1 when cross-presenting antigen to CD8+ T cells renders these T cells unresponsive and establishes a PDL-1 dependent antigen-specific T cell tolerance in the liver (16, 17). Futhermore, as T cells transmigrate across the LSEC barrier to enter the liver parenchyma, the LSECs are able to detect allospecificity and induce T cell death both directly and indirectly via the Fas/FasL pathway (19, 45).

#### Kupffer Cells

KCs are liver-resident, immobile macrophages located within the sinusoidal lumen. They are hugely abundant, constituting 80% of the body's entire macrophage population and around 35% of non-parenchymal cells within the liver (5). KCs have been found to be essential mediators of homeostatic tolerance in the liver. KCs express significantly lower levels of MHC-II and costimulatory molecules compared to dendritic cells, meaning that they are incapable of sufficiently priming T cells on their own. Notably, they can block dendritic cell priming of antigen-specific T cells in a prostaglandin-dependent manner *in vitro* (21). Under steady-state conditions, KCs survey the sinusoids for dead cell debris, pathogens and particulates to phagocytose and this surveillance role can both establish tolerance or rapid response to pathogen depending on the physiological context. KCs phagocytose and present non-pathogen derived antigenic particulate matter and generate a skew in liver CD4+ T cells toward non-responsiveness (22). Heymann et al. shed light on the efficacy of KCs to induce tolerance by tracking OVA-loaded liposomes using intra-vital microscopy. KCs were the primary cell type within the liver to internalize labeled particulates and promoted the expansion of CD25+FoxP3+ OVA-specific Tregs *in vivo*. Both KC depletion and liver inflammation prevented tolerance induction (22).

Their essential sentinel role is further highlighted in mouse models lacking in KCs, where mice are fatally unable to clear a range of bacterial infections (46–48). When encountering pathogen, KCs rapidly release pro-inflammatory cytokines TNF-α, IL-6, and IL-1, promoting the recruitment of granulocytes and neutrophils to clear pathogens (46, 49). Following initial pro-inflammatory function, KCs then express the suppressive mediator IDO and release PGE-2, IL-10, and TGF-β to quench localized inflammation (21, 42, 50).

Targeting KCs to induce antigen-specific tolerance is a promising avenue when considering immunotherapeutic particle delivery for treatment of autoimmune diseases, but would require administration in contexts without liver inflammation. It may therefore not be the most appropriate method for addressing liver autoimmune diseases without prior immune suppressive treatment.

#### Hepatic Stellate Cells

HSCs can also act as APCs and present antigens via MHC-I, MHC-II and CD1d (51). They are powerful producers of TGF-β and retinoic acid within the liver, helping to maintain a generalized immunosuppressive milieu at homeostasis and promoting Treg differentiation and residence within the liver (25, 26). However, when HSCs become activated in the presence of pathogens or strong inflammatory signals, they rapidly metabolize stored Vitamin A and differentiate into myofibroblasts, secreting extra-cellular matrix proteins. Therefore, HSC are key drivers of hepatic fibrosis and associated deterioratio to cirrhosis (52).

#### Hepatocytes

Hepatocytes themselves possess tolerogenic properties, as they are MHC-II expressing in the absence or very low expression of costimulatory molecules (27, 28). In mice, hepatocytes in inflammatory conditions can activate a Notch and IFN-γ dependent pathway to divert Th1 CD4+ T cells to synthesize IL-10 (31). PD-L1 is also inducible in hepatocytes by viral infection and by type 1 and type II interferons, mediating apoptosis of activated T cells (29).

At present, it is unclear exactly which of these tolerance-promoting mechanisms fail in the pathogenesis of autoimmune liver diseases, and at which time in disease progression. The consequence of these homeostatic mechanisms failing, however, can be devastating for liver function, impairing tissue regeneration and causing fibrosis. In the case of autoimmune liver diseases, immunological targeting of liver self-antigens catalyzes a system of inflammation and chronic liver disease. It will be important to understand which mechanisms break down in the process of developing autoimmune liver disease, in order to best intervene with tolerance promoting treatments.

## Autoimmune Liver Disease

**Figure 2:**
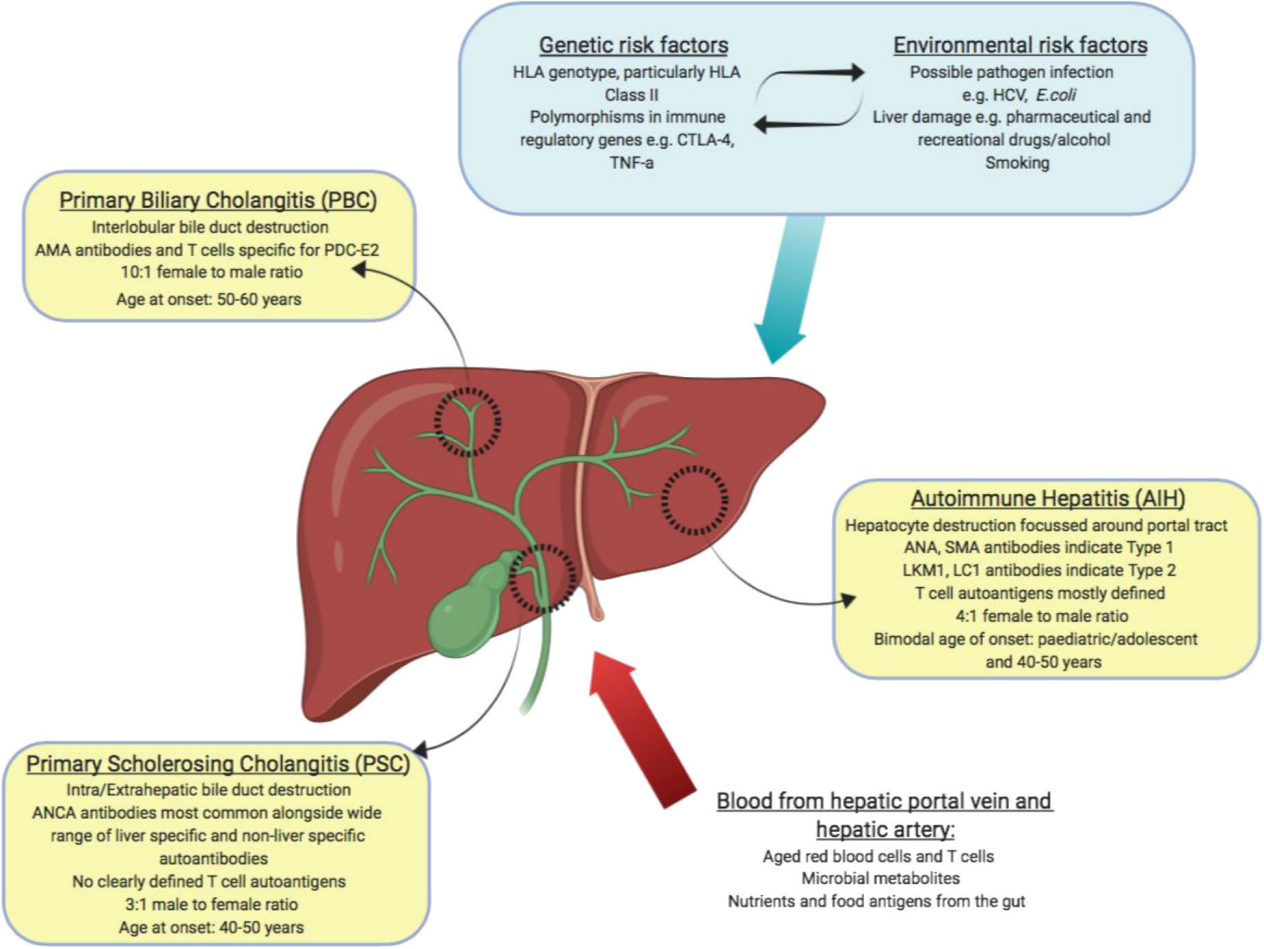
Summary of autoimmune liver diseases: tissues affected, and key features of disease (yellow boxes). Blood borne factors which challenge the maintenance of immune tolerance are listed as inputs (red arrow). Genetic, environmental and lifestyle factors which could affect the maintenance of tolerance are listed as inputs (blue box and arrow).

Autoimmune liver disease (AILD) can be divided into 3 distinct clinical diseases, autoimmune hepatitis (AIH), primary biliary cholangitis (PBC) and primary sclerosing cholangitis (PSC). They are distinguished by the molecular and cellular targets of immune pathology alongside the location of observed liver damage (Figure 2). Biliary dominant PBC and PSC affect cholangiocytes lining bile ducts. PBC destroys small, interlobular bile ducts while PSC targets larger bile ducts and is characterized by inflammatory fibrosis in the intrahepatic and extrahepatic biliary tree (53, 54). In AIH, the target is hepatocytes themselves, leading to interface hepatitis and significant lymphocyte infiltration primarily around the portal tracts (55). All 3 diseases will develop to severe liver fibrosis without medical intervention.

## Disease Characteristics and Epidemiology

AIH is a chronic progressive liver-disease that mainly affects women (70–80% cases) and can be diagnosed in adults and children of any age or ethnicity (56). As symptoms and biochemical indicators are widely heterogenous between patients, the International Autoimmune Hepatitis Group (IAIHG) developed a scoring system based on specific criteria to improve early diagnosis (57, 58). Early diagnosis is imperative as cirrhosis is already present at diagnosis of a third of AIH patients and liver cirrhosis is the primary risk factor associated with development of hepatocellular carcinoma (56). AIH is a rare disease affecting between 16 and 20 cases per 100,000 (59–62) but appears to be increasing in prevalence. A long-term Danish study observed an almost 2-fold increase in the annual incidence rate of AIH between 1994 and 2012 (63).

PBC affects around 35/100,000 individuals, and is most common in women (9:1 female: male) and those over 50 years old (64). Reports have also indicated increasing prevalence of PBC over time (65). In around 10% of PBC patients, there will be overlap disease with features of AIH (66).

PSC is lowest in prevalence, and most commonly found in Northern Europe with 5.5–8.5 patients per 100,000 individuals in the UK, which has increased by about 50% since 1991 (67, 68). Unlike AIH and PBC, PSC is more common in men than in women (3:1) and although disease can occur at any age it has a peak incidence around 40 (69).

## Genetic Associations

There is evidence for genetic factors playing a role in pathogenesis of all 3 AILD disease classes, with both major histocompatibility complex HLA genes and non-HLA genes showing disease associations (70). Exactly how HLA confers increased disease risk is unknown, but is presumed to be related to how antigens are presented and recognized by the immune system.

AIH-1 usually presenting in middle age has been linked to HLA-DRB1^*^0301 and HLA-DRB1^*^0401 with co-expression of these risk alleles indicating a double-dose effect (71–73). AIH-2 affects around 10% of AIH patients, exhibits a more aggressive phenotype and has been related to the presence of HLA-DRB1^*^07 and DRB1^*^03 in cohorts in the UK and Brazil (74). AIH-2 is most commonly diagnosed in childhood and has even been recorded in infants, suggesting a potentially different etiology to AIH1 (75). Around 20% of AIH patients suffer concomitant autoimmune diseases, most commonly thyroiditis (also HLA-DR3), inflammatory bowel disease (IBD), Type 1 diabetes (also HLA-DR4/HLA-DR3) and Addisons disease (HLA-DR4) (76).

PBC susceptibility is highly associated with HLA-DRB1^*^08 in Europe and North America (77). In contrast HLA-DRB1^*^11 and HLA-DRB1^*^13 were found to be protective toward PBC (78).

PSC is generally associated with HLA-DRB1^*^0301 in Norwegian and British patients (79). In patients with both PSC and inflammatory bowel disease, PSC is also associated with HLA-DRB1^*^13 but only with individuals with IBD (80).

GWAS studies have also identified association between specific polymorphisms within regulatory genes and AIH, PBC, PSC development. Notably for AIH and PBC but not for PSC, these include CTLA-4 and TNF-α genes (81–86) which are identified in similar studies of wide ranging autoimmune disorders (87, 88). TNF-α is located in the HLA-DR/DP locus; therefore, its appearance in GWAS studies of autoimmune diseases is unsurprising. However, at present it is unclear whether its influence is merely by association via linkage disequilibrium, or whether its function and downstream signaling actively contributes to the strong correlation of certain HLA haplotypes to autoimmunity. A further interesting correlation between TNF-α and CTLA-4 noted that single nucleotide polymorphism (SNP) rs1800629 of the TNF-α gene, leading to increased TNF-α production, amplified the CTLA4 SNP risk associated with rs231725, and that the combination of both SNPs was significantly more common in PBC patients compared to healthy controls (84). Studies in PBC have also identified common variant in IL-12 and IL-12R which indicate a role for aberrant IL-12 signaling in disease pathogenesis (89).

The specific triggers that lead to development of AILD are as yet poorly understood, due to the complex nature of genetic and environmental (drug and foreign pathogen) influences. It is thought that environmental stressors on a background of genetic predisposition in the form of HLA haplotypes and general tolerogenic “fitness” (Tregs and feedback loops) could help establish chronic autoimmune liver injury. AILD patients commonly present other autoimmune diseases, suggesting that immune dysregulation is not isolated to the liver in these cases.

## Autoantigens and Autoantibodies

Characteristic of all autoimmune diseases, AIH and PBC have autoantibodies present in patient's circulation. In both diseases, there are a some well-defined autoantibodies that are used to diagnose patients; however, the autoantigens that these antibodies are specific for is less well-defined. In contrast, PSC patients do not possess defined liver-specific autoantibodies. The strongest biomarker associated with PSC is elevated serum alkaline phosphatase levels, indicative of cholestasis (69, 90). PSC is usually diagnosed by MRI imaging of the biliary tree to identify cholestasis and/or strictures (69, 91, 92). Up to 80% of PSC patients also present with inflammatory bowel disease (IBD), indicating a general gastrointestinal inflammatory phenotype (93). Taken alongside the fact that PSC is more common in men and has less strong HLA associations, the lack of known autoantibodies calls into question whether the disease is strictly autoimmune, or whether it is autoinflammatory in nature (94).

Suspected AIH patients are scored according to International AIH Group published criteria to determine a diagnosis of AIH (57, 58). For clinical and research purposes, patients are grouped into AIH-1 or AIH-2 by the presence of different autoantibody profiles to liver antigens. The definitive clinical distinction between AIH subtypes is challenging, and age-matched patients usually follow similar trajectories and treatment protocols regardless of patient autoantibody profiles (95).

The vast majority (≈75%) of AIH-1 patients are positive for anti-nuclear antibodies (ANA) and/or anti-smooth muscle antibodies (SMA) (62, 63). However, these autoantibodies are not limited to AIH-1 patients and the autoantigens responsible are not well-defined (96, 97). ANA can react to histones, ribonucleoproteins ds-DNA and chromatin (98). SMA also have a range of specificities, predominantly to F-actin (99, 100). The remainder of patients who lack ANA or SMA antibodies, but present with liver disease pathology in accordance with the IAIHG diagnosis criteria, may possess other defined autoantibodies including anti-perinuclear neutrophil cytoplasmic antibodies (pANCA), anti-liver cytosol (LC-1), anti-soluble liver antigen/liver-pancreas (SLA/LP) and/or asialoglycoprotein receptor (ASGPR). Of note, SLA/LP is present in around 30% of AIH patients and has been identified in both adults and children (101–103). SLA/LP autoantibodies are specific to the autoantigen SLA/LP/tRNP(Ser)Sec (104, 105) and is therefore the only defined autoantigen implicated in AIH-1.

AIH-2 is rarely seen as a newly-diagnosed disease in adult cohorts but is reported to represent around 30% of pediatric AIH patients (106). AIH-2 has a less varied autoantigen profile and is diagnosed predominantly by the presence of anti-liver kidney microsomal antibody (LKM-1) and to a lesser extent anti-liver cytosol antibody (LC-1), specific to the liver proteins cytochrome P450 2D6 (CYP2D6) and formiminotransferase cyclodeaminase (FTCD), respectively (74, 107). Both T cell and B cell epitope mapping studies of CYP2D6 have been published, providing evidence that CYP2D6-reactive lymphocytes circulate in AIH-2 patients but not in healthy people (74, 108). Again, neither LKM-1 or LC-1 autoantibodies are restricted to AIH-2 – notably LKM-1 antibodies are detected in 5–10% of chronic HCV patients (101, 109) with an identified homologous sequence between HCV and CYP2D6 judged to be the cause (102).

The success of antigen-specific immunotherapies in re-establishing tolerance is reliant on having strong knowledge of the autoantigens underpinning immune pathology. Therefore, with our current understanding of AIH disease, it is likely that the most appropriate immediate targets for AIH-2 are CYP2D6, FTCD and for AIH-1 SLA/LP/tRNP(Ser)Sec. To be applicable to the majority of AIH-1 patients, however, detailed antigen profiling of AMA and SMA targets is required but has proved to be extremely challenging thus far.

PBC is diagnosed by the presence of highly-specific anti-mitochondrial antibodies (AMA) against the pyruvate dehydrogenase complex (PDCE2) (110–112). Over 90% of PBC patients are positive for AMA antibodies (113). PDCE2 is expressed at detectable levels on biliary epithelial cells in PBC but not in healthy individuals (114, 115). A minority of PBC patients are AMA negative, however, histological analyses of the bile ducts reveal no difference in pathology and presentation of PDCE2 between AMA positive and AMA negative PBC patients (114). Interestingly, PBC is also associated with prior urinary tract infections which are most frequently caused by *E.coli* (116–118). It is thought that *E.coli* induces B and T cell cross reactive responses to human PDCE2 by molecular mimicry (115).

In the case of AIH and PBC the presence of reliable autoantibodies to known autoantigens, and lymphocytes specific to these autoantigens found in patients provides vital evidence that supports targeting autoreactive cells in patients could have therapeutic benefit.

## Current Treatments

The clinical options to treat AILDs are limited once diagnosis is confirmed. The current front-line treatments center on broad immunosuppressive agents and ursodeoxycholic acid (UDCA) – a biliary protective drug of which the mechanism of action is still poorly understood.

In AIH, randomized controlled trials from the 1970's helped establish the mainstay treatment options of corticosteroids (PRED) and azathioprine (AZA) (119–121). Today, 50 years later, the treatment plan is almost identical to these early trials. This is sufficient to obtain biochemical disease remission and to prevent further liver damage in around 80% of AIH-1 patients (122). However, this level of immunosuppression commonly causes side effects including Cushingoid features, weight gain and gastrointestinal issues. For the vast majority of patients immunosuppressive therapy is lifelong, bringing a range of side effects, including osteoporeosis (especially problematic in middle aged women), diabetes mellitus, an increased risk of infections and risk of both hepatocellular and extra-hepatic cancers (123). Despite treatment, *de novo* cirrhosis occurs in around 14% of patients increasing the likelihood patients progress to transplant or hepatocellular carcinoma (124, 125). Adolescents often display poor treatment regime compliance, leading to the highest rate of relapse of any age group; therefore, an approach which causes fewer side effects, would be particularly welcome in this cohort (126). A recent trial using the corticosteroid budesonide with AZA indicated improved efficacy to PRED and a much improved adverse effect profile (127). So far, this is yet to be translated to a change in clinical treatment practices for AIH.

The primary course of treatment for PBC is UDCA (128). UDCA slows PBC disease progression by protecting cholangiocytes and hepatocytes from damage (129). UDCA significantly improves transplant free survival (130, 131); however, up to 40% of patients treated with UDCA have an insufficient response to treatment (132, 133), therefore in the long term, a liver transplant is often required. Even with a liver transplant, PBC recurs in around 30% of patients after 10 years (134–136). A recent development in approved PBC treatment is administration of obeticholic acid, particularly in patients refractory to or intolerant of UDCA. Obeticholic acid significantly improved liver function tested by alkaline phosphatase levels in patients with insufficient UDCA responses, with 69% of treated patients achieving a 20% reduction in ALP vs. only 8% of patients treated with UDCA alone (137, 138).

There are no effective treatments for PSC that have been proven to improve transplant free survival. There is no clear evidence that UDCA can treat PSC despite multiple clinical trials (139, 140). Trials applying other immunosuppressants to PSC, including prednisolone, budesonide, azathioprine, cyclosporin, methotrexate, mycophenolate, and tacrolimus have not shown efficacy (141). Drugs that antagonize the effects of anti-TNF-α such as pentoxifylline, etanercept and anti TNF-α monoclonal antibodies are also ineffective (141). Patients may undergo several of these pharmacological interventions in an attempt to quench biliary pathology, yet for most the only long-term option is liver transplantation. The mean time from diagnosis to liver transplantation/death is 9–12 years (90, 142). Unfortunately, PSC is expected to reoccur in 20–25% of patients over a 5–10 year period (136, 143, 144).

There is certainly an unmet need for improved treatment options with increased efficacy in hard to treat groups particularly pediatric AIH patients, refractory PBC patients and PSC patients. With the current understanding of the features of PSC, it is not clear that its pathogenesis is autoimmune, thus without the identification of autoantibodies and autoantigens relevant to PSC it will not be possible to generate antigen-specific immunotherapies for these patients. For AIH and PBC patients, however, there is sufficient evidence that antigen-specific immunotherapies could have real therapeutic value, and in contrast to systemic immunosuppressive drugs these should have a more specific mechanism of action that does not threaten the general health and immune capacity of the patient. The need for antigen-specific immunotherapies becomes ever more important as the world faces highly infectious agents such as the SARS-CoV-2 virus: such pathogens clearly endanger anyone taking immunosuppressive drugs.

## Antigen-specific Immunotherapy

**Figure 3:**
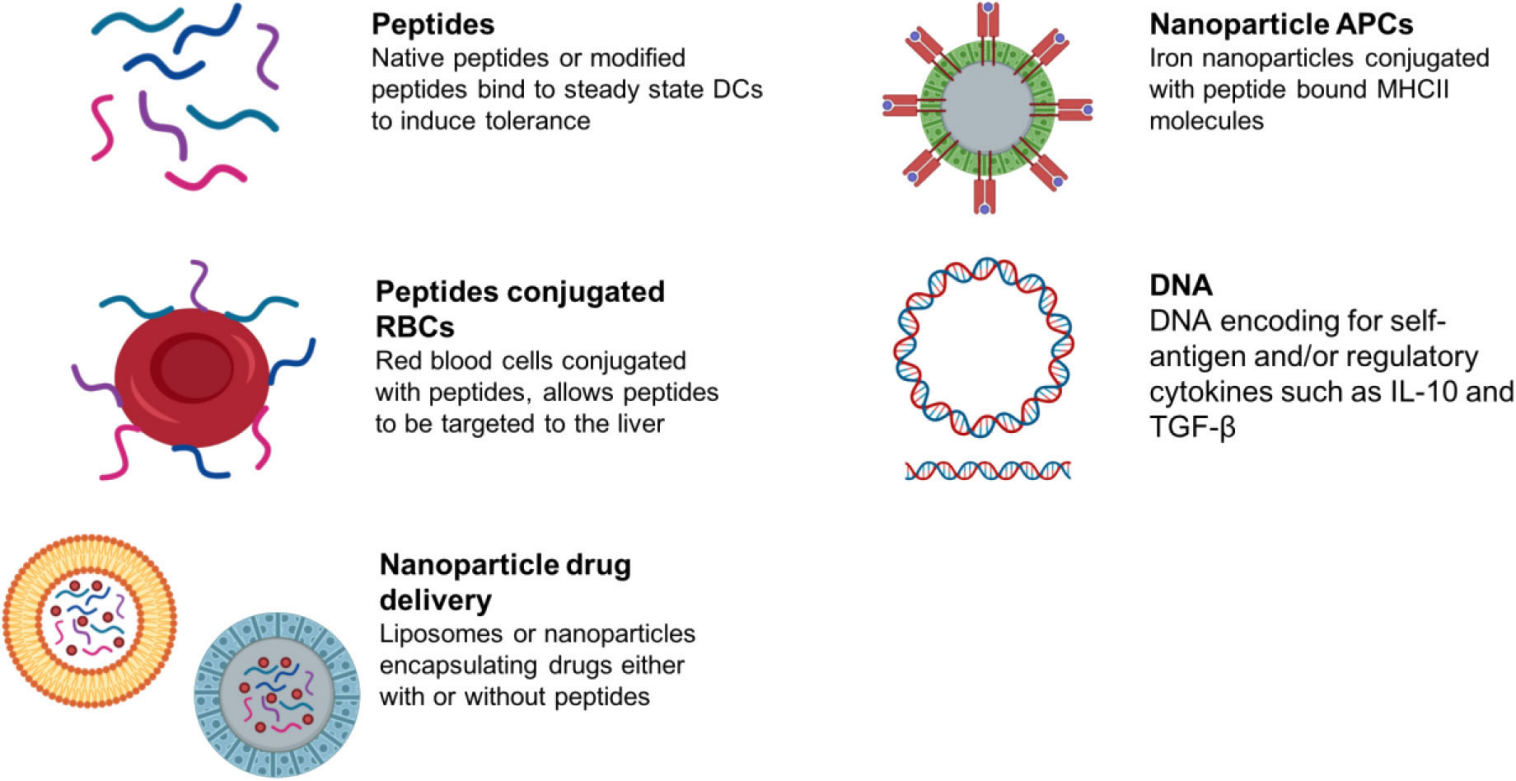
Summary of antigen-specific immunotherapy approaches in preclinical/clinical development.

Antigen-specific immunotherapy has been practiced in the field of allergy for more than 100 years (145, 146). Recently, there has been increasing interest in the development of antigen-specific approaches for specific immunotherapy of autoimmune conditions (schematic summary in Figure 3). This follows evidence that treatment of experimental animals with antigens can lead to amelioration of disease (146). Currently these approaches target CD4 T cell recognition of self-antigens. This is because CD4 T cells control the generation of all of the tissue damaging mechanisms associated with autoimmunity including pathogenic autoantibodies, antigen-driven inflammation and self-antigen specific CD8 T cells. It is not the focus of this review to discuss the mechanisms of action underpinning each approach aiming to induce antigen-specific tolerance; as these has been described comprehensively recently elsewhere (147, 148). We have briefly summarized within Table 2 the proposed mechanisms of action for each approach in development or in the clinic.

**Table 2:** This table summarizes the current status of pre-clinical and clinical developments of antigen-specific immunotherapies for autoimmune diseases.

**Company**	**Delivery approach**	**Proposed mechanism of action**	**Impact on T cell response**	**Efficacy in experimental models**	**Clinical trials progress**
Anokion	Antigens modified with polymeric forms of either N-acetylgalactosamine or N-acetyl-glucosamine	Target hepatic antigen-presenting cells	Induce CD4+ and CD8+ T-cell deletion and anergy	EAE^A^, T1D	Enrolling patients for KAN-101 trial in coeliac disease
Apitope International NV	Synthetic peptides designed as antigen processing independent CD4+ T cell epitopes (apitopes) injected in saline i.d. or s.c.	Highly soluble peptides traffic to and selectively bind to MHC II antigens on steady-state DC in lymphoid organs	Induction of anergy and generation of regulatory T cells (primarily Tr1)	EAE and Graves' disease models (149, 150)	Phase Ia in SPMS (149) Phase Ib in RRMS (151) Phase II in RRMS (151) Phase I in Graves' disease (152)
Cellerys	Red blood cells (RBC) coupled with peptides from myelin in MS	RBC target macrophages and Kupffer cells in spleen and liver	Increase in Tr1 cell response to antigen with reduced IFN-γ		Phase 1 in RRMS^B^
Cour/takeda	Antigen encapsulated in PLG [poly(lactide-co-glycolide)] nanoparticles	Ag-PLG internalized by splenic marginal zone macrophages and liver phagocytic cells via scavenger receptors (MARCO)	Increase in Foxp3 Treg cells, dependent on CTLA-4, PD-1 and IL-10	EAE, T1D and coeliac disease models (153–155)	Phase I trial of gliadin-PLG in patients with coeliac disease (unpublished)
Dendright/Janssen Biotech Inc	Antigen with calcitriol in liposomes	Liposomes (105–135 nm) target steady-state DC in draining lymph nodes	Increase in Foxp3 Treg cells	Autoimmune arthritis and experimental Goodpasture's vasculitis (156)	Phase I in ACPA+ rheumatod arthritis^C^
Imcyse	T cell epitopes modified by addition of a thioredox motif (CXXC), injected in Alum adjuvant	Promotes cytotoxic activity in T cells through increasing expression of granzyme B and FasL	Cytotoxic cells delete B cells in cognate recognition	T1D (157)	Phase I with 3 staggered doses of modified pro-insuln peptide in T1D (unpublished)
Novo nordisk	Plasmid DNA encoding proinsulin and co-expressing IL-10 and TGF-β	Promotes treg cells	Promotes Treg cell differentiation	T1D with vector expressing GAD antigen (158)	
Parvus	Nanoparticles coated with MHC II proteins and antigenic peptides	Bind directly to CD4^+^ effector cells	Drives differentiation of Tr1 cells from Th1 precursors in mice	EAE, CIA, T1D and autoimmune liver diseases (159, 160)	In pre-clinical development for T1D and autoimmune liver diseases
Selecta	PLG nanoparticles containing rapamycin co-administered with antigen	Nanoparticles found in dendritic cells in spleen and LSEC and Kupffer cells in the liver where they mediate down-regulation of CD80, CD86, class II MHC and upregulation of PDL-1	Promotes Treg cell differentiation	EAE and anti-drug antibodies (161, 162)	Phase II study in gout designed to block the anti-drug antibody response to Pegadricase^D^
Tolerion	DNA encoding self-antigen	CpG islands in DNA replaced with GpG to reduce immunogenicity of antigen delivery	Promote immune regulatory response to self-antigen	BHT-3021 prevents T1D in mouse model (163)	Phase I trial completed and phase II enrolling (164)
Topaz	Ferromagnetic nanoparticles coupled to T cell epitopes	Nanoparticle-based autoantigen delivery to liver sinusoidal endothelial cells	Induction of Foxp3^+^ Treg cells in the liver	EAE (165)	First patient enrolment in phase I trial of TPM203 in Pemphigus Vulgaris

*Where either pre-clinical or clinical trials have been published these are referenced. Additional results are discussed in relevant conference abstracts and company websites*.

A *https://anokion.com/wp-content/uploads/2019/09/ECTRIMS_Poster_9.13.19.pdf*.

B *MULTIPLE SCLEROSIS JOURNAL Volume: 25 Special Issue: SI Supplement: 2 Pages: 894–894 Meeting Abstract: 339 Published: SEP 2019*.

C *https://acrabstracts.org/abstract/a-phase-i-randomized-double-blind-placebo-controlled-single-center-single-dose-escalation-to-investigate-the-safety-tolerability-and-pharmacodynamics-of-subcutaneously-administered-den-181-in-a/*.

D *https://selectabio.com/immtor/gouttherapy/phase2results*.

Allergic desensitization involves administration of increasing and repeated doses of allergen, often a crude extract of the allergen material. Early attempts to treat autoimmune diseases in a similar way were not successful with intact antigen inducing pathogenic autoantibodies (166, 167) or driving tissue damaging cytotoxic T cells (168). To ensure safety and efficacy, autoantigens must be modified in such a way as to protect the recipient from exacerbation of the autoimmune response or they must be fragmented so as to avoid engagement with pathogenic autoimmune mechanisms. A preferred approach is to use short fragments of antigens (synthetic peptides) designed to modulate CD4 T cells but lacking either the structural integrity to engage pathogenic B cells or the peptide sequences to engage CD8 T cells.

It is important to appreciate that the mammalian adaptive immune system is poised to respond to foreign antigens but in the steady-state is adapted to limit autoimmune responses to the individual's own antigens. Responsibility for distinguishing between self and foreign antigens falls primarily on dendritic cells (169). In the steady-state, these cells are capable of binding the many fragments of self-antigens that are contained within the lymphoid pool (170). Steady-state dendritic cells presenting self-antigens are tolerogenic. It is only when these cells encounter foreign antigens in the context of microbial pattern-associated molecular patterns (e.g., LPS, bacterial DNA etc.) that they present antigen in an immunogenic rather than a tolerogenic fashion.

There are now a variety of clinical trials in progress that target steady-state/immature dendritic cells either *in vivo* or *in vitro*. The *in vitro* approach involves the generation of myeloid-derived dendritic cells treated with immunosuppressive agents, such as vitamin D3, to maintain a tolerogenic phenotype. The cells are then treated with peptides from self-antigens and reinjected into the patient (171–173). Alternatively, antigens can be coupled to dendritic cell targeting antibodies (e.g., anti—Dec205) for *in vivo* targeting (174). Our own work has focused on designing peptides that target steady-state dendritic cells directly. Early studies showed that some but not all known CD4 T cell epitopes induce tolerance when injected into experimental mice (175). Peptides must bind directly to MHC Class II and adopt the same conformation as the naturally processed epitope (176). Those peptides that do not mimic the naturally processed epitope fail to induce tolerance in relevant T cells. This implies that tolerogenic peptides bind directly to MHC Class II on or in steady state dendritic cells without further processing. Recent work from our laboratory has shown that such antigen-processing independent epitopes (apitopes) selectively bind to peptide receptive MHC class II molecules on steady-state dendritic cells but not to MHC Class II on the surface of B cells or monocytes. This is explained by the distinct, peptide-receptive nature of MHC Class II molecules on steady-state dendritic cells (177). Furthermore, tolerogenic peptides are detectable on steady state DCs up to 5 days after administration (178). We have shown that apitopes induce tolerance by induction of anergy in self-antigen reactive T cells and the expansion of antigen-specific Tr1 cells (179–182).

Alternative approaches for targeting “tolerogenic” APCs *in vivo* include combining antigen with liposomes, red blood cells or nanoparticles (Summarized in Table 2). These target different antigen-presenting cells in lymphoid organs or the liver depending on the size of the material or nanoparticle. This determines their modus operandi.

There is increasing evidence that nanoparticles of different sizes transit to and are taken up by different APCs according to their size. Berkland et al. have shown that particles > 200 nm are retained in the liver while those <4 nm are rapidly excreted (183). This evidence would pair well with evidence from Kupffer cell studies that these cells establish tolerance by phagocytosing particulate material and presenting antigenic fragments (21, 22). Such small particles rapidly drain from sites of injection into blood and lymph and particles of 4–10 nm penetrate lymph node cortex where they can interact with steady-state DCs. In contrast, particles > 100 nm are retained in the sub-capsular space where they will be processed by macrophages.

Santamaria et al. have developed artificial APCs (Navacims) based on nanoparticles coated with MHC Class II and antigenic peptide (159). The mechanism of action is in principle the same as apitope immunotherapy, both establish immunological tolerance by inducing IL-10 expressing CD4 T cells through a negative feedback mechanism (159, 160, 181, 184, 185). The resulting Tr1 cells are characterized by the expression of the immunosuppressive genes such as IL10 and co-inhibitory receptors (186, 187). The Tr1 cells induced by Navacims, however, also express inflammatory cytokines such as TNF-α, IL5, and GM-CSF (188). In contrast, Tr1 cells derived from apitope immunotherapy do not express TNF-α, IL5, or GM-CSF (182). Their recent studies serve as a valuable proof of concept, as antigenic peptides identified by *in silico* binding predictions from PDC-E2 loaded onto IAg7 MHC-nanoparticles are able to ameliorate PBC-like liver damage.

These Navacims, MHCII-based nanomedicines displaying epitopes from mitochondrial, endoplasmic reticulum, or cytoplasmic antigens associated with primary biliary cholangitis or autoimmune hepatitis can suppress disease progression in various murine models in an organ- rather than disease-specific manner (160). The improvement in liver score was shown to be IL-10 and TGF-β dependent. However, none of these liver disease models fully recapitulates the human condition. Furthermore, the T cell epitopes restricted by murine MHC class II molecules are unlikely to resemble those binding HLA-DR and DQ molecules i.e., relevant to human disease

However, Navacims do not work prophylactically to prevent disease onset, this is in contrast to apitope immunotherapy which is effective before as well as after disease onset (160, 179, 182). The bystander suppression demonstrated by loading the artificial APCs with PDC-E2 peptides and supressing the response against the CYP2D6 antigen and *vice versa* is intriguing and suggests that bystander suppression can influence different autoimmune conditions within the same tissue (189).

## Future Prospects for Antigen-specific Immunotherapies for Autoimmune Liver Diseases

At this stage, it is too early to compare the safety and efficacy of the various approaches shown in Table 2. It is likely that different approaches will prove more or less effective for control of different immune pathologies and diseases. It is of paramount importance, however, to apply three tests to these approaches.

1. What is the mechanism of action? It will be critical to fully understand the mechanism by which these approaches induce antigen-specific tolerance both in experimental models and in patients.
2. Which approaches induce bystander suppression? For diseases like Graves' disease, we know precisely what the target antigen is. However, for most autoimmune diseases we do not know which antigen is targeted by the immune system to initiate the disease. For many others, antibodies specific for self-antigens are associated with disease but may or may not have a role in immune pathology. Furthermore, in most autoimmune conditions, epitope spreading leads to the generation of an immune response to a range of antigens within the same tissue (190). In order to account for epitope spreading, we and others have shown that certain immune regulatory mechanisms, such as Tr1 cells, mediate bystander suppression (191). By targeting antigen A within a tissue and eliciting immunosuppressive regulatory T cells, we can control the immune response to antigens B, C, D etc. within the same tissue.
3. Which approach permits repeated antigen administration? Apitope has now conducted clinical trials in multiple sclerosis and Graves' disease. In both cases, protection from immune pathology was observed but the patients treated did not enter a permanent state of tolerance (149, 151). Protection was seen for up to 1 month after the last dose of peptide which correlates well with the duration of tolerance observed in euthymic mice (192). It may well be that humans have evolved to require continued exposure to antigens in order to maintain tolerance. For this reason, it is likely that repeated administration of the different tolerogenic materials described in Table 2 will be required. A successful therapeutic approach must avoid induction of anti-drug antibodies or non-specific immune suppression.

There is already substantial progress in the quest for specific immunotherapies for autoimmune liver diseases. With this in mind, our laboratory is designing putative disease-altering apitopes from the dominant human autoantigens associated with PBC and type 2 AIH.

## Author Contributions

All authors listed have made a substantial, direct and intellectual contribution to the work, and approved it for publication.

## Conflict of Interest

DW is Professor of Immunology at the University of Birmingham and CSO and Founder of Apitope International NV. The remaining authors declare that the research was conducted in the absence of any commercial or financial relationships that could be construed as a potential conflict of interest.
